# Microscopic alterations of the gastric mucosa in preneoplastic lesions as assessed by new-generation endocytoscopy

**DOI:** 10.1055/a-2119-1212

**Published:** 2023-08-21

**Authors:** Riccardo Vasapolli, Lukas Neuhaus, Jörg Schirra, Jens Neumann, Julia Mayerle, Peter Malfertheiner, Christian Schulz

**Affiliations:** 1 Medical Department II, University Hospital of the Ludwig Maximilians University Munich, Munich, Germany; 2 DZIF Deutsches Zentrum für Infektionsforschung, Partner Site Munich, Germany; 3 Institute of Pathology, Faculty of Medicine, Ludwig Maximilians University of Munich, Munich, Germany

Chronic atrophic gastritis with/without intestinal metaplasia is defined as a gastric precancerous condition (GPC) that increases the risk of gastric cancer according to the severity of atrophy 1 . Advanced endoscopic imaging permits accurate visualization of the mucosa and optical diagnosis of GPC 2 . Endocytoscopy introduces a further step, providing ultrahigh magnification images and in vivo histologic assessment of nuclear and cellular structures; however, data on endocytoscopic characterization of GPC are lacking 3 .

Here, we report a series of four exemplary cases demonstrating characteristic changes in endocytoscopy along different histological stages of gastric carcinogenesis, from chronic nonatrophic gastritis through atrophic gastritis, intestinal metaplasia, and gastric dysplasia/adenocarcinoma ( Video 1 ).

**Video 1** Endocytoscopic characterization of gastric mucosa in patients with 1) chronic nonatrophic gastritis, 2) chronic atrophic gastritis, 3) intestinal metaplasia, and 4) gastric dysplasia/adenocarcinoma. Examinations were conducted using an Olympus endoscopy system (GIF-H290EC – Evis X1 CV-1500; Olympus, Tokyo, Japan) after double staining of the gastric mucosa with 0.05 % crystal violet and 1 % methylene blue.


Patient 1 was a 58-year-old woman who underwent esophagogastroduodenoscopy because of dyspeptic symptoms (no alarm symptoms). White-light endoscopy (WLE) showed diffuse erythema of the gastric mucosa without focal lesions. Histology confirmed the diagnosis of a mild *Helicobacter pylori* -negative chronic nonatrophic gastritis ( Fig. 1 ). A 4-week course of proton pump inhibitor therapy led to improvement of the symptoms.

**Fig. 1:**
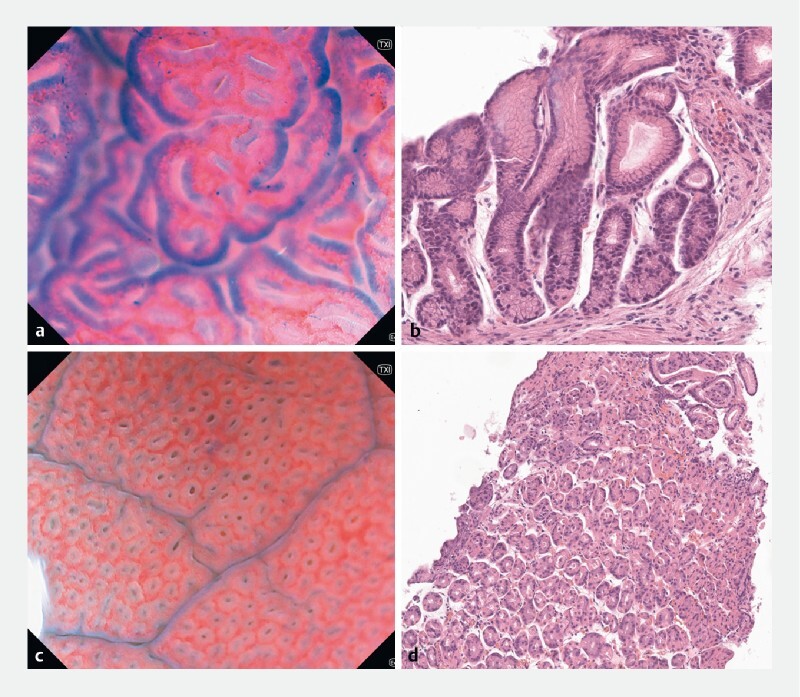
Images from patient 1 with chronic nonatrophic gastritis. **a** Regular arrangement of foveolar structures with a few scattered, small, and poorly stained nuclei (antrum ~ × 350). **b** The corresponding histopathological picture. **c** Homogeneously distributed and regular gastric pits delineated by a consistent honeycomb-like subepithelial capillary network (corpus, ~ × 200). **d** The corresponding histopathological picture.

Patient 2 was a 46-year-old woman with autoimmune gastritis who was referred for surveillance endoscopy. WLE revealed multifocal atrophy, which was severe (stage III) according to the histopathological operative link on gastritis assessment (OLGA) staging system 4 ( Fig. 2 ).

**Fig. 2:**
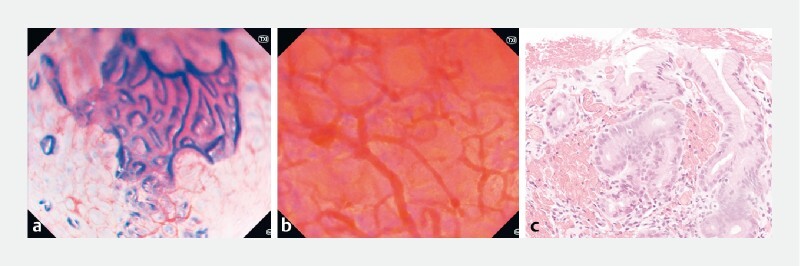
Images from patient 2 with chronic atrophic gastritis. **a** Patchy distribution of atrophic areas, with subtotal loss of the glandular pattern (corpus, ~ × 300). **b** Deformation of gastric pits, depletion of marginal epithelium with reduced dye uptake that revealed multiple translucent vessels (corpus, ~ × 520). **c** The corresponding histopathological picture.

Patient 3 was a 48-year-old man with alcoholic liver cirrhosis who underwent index esophagogastroduodenoscopy that revealed small esophageal varices. There were no signs of portal hypertension in the stomach but multiple areas of intestinal metaplasia were present. The operative link on gastric intestinal metaplasia (OLGIM) stage 4 was III ( Fig. 3 ).

**Fig. 3:**
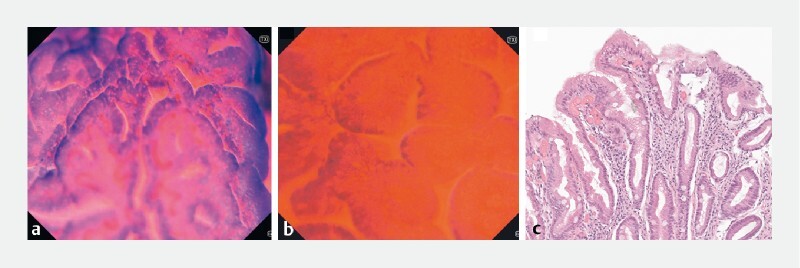
Images from patient 3 with chronic atrophic gastritis and intestinal metaplasia. **a, b** Compact arrangement of glandular structure with narrowing of the lumens (slit-like lumens), increased dye uptake, and detection of multiple goblet cells (antrum, ~ × 300 [ **a** ], ~ × 520 [ **b** ]). **c** The corresponding histopathological picture.

Patients 2 and 3 were included in a 3-year surveillance program in accordance with current guidelines 5 .

Patient 4 was a 70-year-old man with hepatic metastases. WLE revealed a 3-cm flat ulcerated lesion on the anterior wall of the gastric corpus. Histopathological analysis of targeted biopsies confirmed atrophic gastritis with advanced GPC (OLGA/IM stage IV) and a moderately differentiated intestinal-type adenocarcinoma in the perilesional and tumor areas, respectively ( Fig. 4 ). The patient received palliative chemotherapy.

**Fig. 4:**
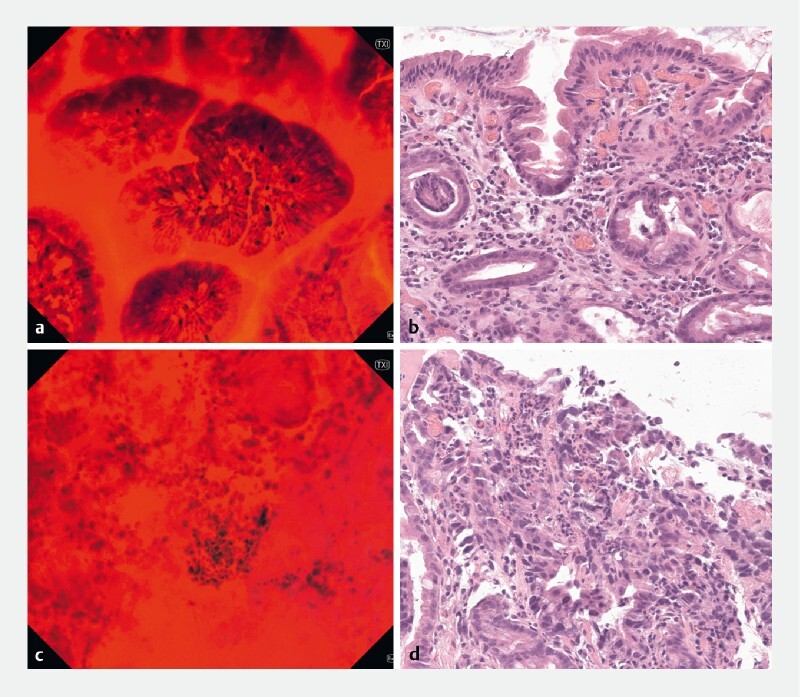
Images from patient 4 with gastric dysplasia/adenocarcinoma. **a** Coexisting features of severe gastric atrophy (i. e. partial loss of glandular structures, reduced dye uptake) and intestinal metaplasia (i. e. lobulated structure, presence of goblet cells) in the perilesional area (distal corpus, ~ × 520). **b** The corresponding histopathological picture (perilesional). **c** Complete disarrangement of gastric foveolae with malignant cytologic characteristics (i. e. hyperchromatic and pleomorphic nuclei, increased nuclear/cytoplasmic ratio) in the tumor area (distal corpus, ~ × 520). **d** The corresponding histopathological picture (tumor).

Endocytoscopy has the potential to support GPC identification reliably and should be further evaluated in upper gastrointestinal diseases.

Endoscopy_UCTN_Code_TTT_1AO_2AB

## Notes

Endoscopy E-Videos

https://eref.thieme.de/e-videos
*E-Videos* is an open access online section of the journal *Endoscopy* , reporting on interesting cases and new techniques in gastroenterological endoscopy. All papers include a high-quality video and are published with a Creative Commons CC-BY license. Endoscopy E-Videos qualify for HINARI discounts and waivers and eligibility is automatically checked during the submission process. We grant 100% waivers to articles whose corresponding authors are based in Group A countries and 50% waivers to those who are based in Group B countries as classified by Research4Life (see: https://www.research4life.org/access/eligibility/ ). This section has its own submission website at https://mc.manuscriptcentral.com/e-videos

## References

[1] Malfertheiner P Megraud F Rokkas T Management of Helicobacter pylori infection: the Maastricht VI/Florence consensus report Gut 2022 10.1136/gutjnl-2022-327745 22491499

[2] East J E Vleugels J L Roelandt P Advanced endoscopic imaging: European Society of Gastrointestinal Endoscopy (ESGE) Technology Review Endoscopy 2016 48 1029 1045 27711949 10.1055/s-0042-118087

[3] Angeli Abad M R Shimamura Y Fujiyoshi Y Endocytoscopy: technology and clinical application in upper gastrointestinal tract Transl Gastroenterol Hepatol 2019 5 28 10.21037/tgh.2019.11.12 PMC7063519 32258532

[4] Capelle L G de Vries A C Haringsma J The staging of gastritis with the OLGA system by using intestinal metaplasia as an accurate alternative for atrophic gastritis Gastrointest Endosc 2010 71 1150 1158 20381801 10.1016/j.gie.2009.12.029

[5] Pimentel-Nunes P Libânio D Marcos-Pinto R Management of epithelial precancerous conditions and lesions in the stomach (MAPS II): European Society of Gastrointestinal Endoscopy (ESGE), European Helicobacter and Microbiota Study Group (EHMSG), European Society of Pathology (ESP), and Sociedade Portuguesa de Endoscopia Digestiva (SPED) guideline update 2019 Endoscopy 2019 51 365 388 30841008 10.1055/a-0859-1883

